# Cushing’s Disease Manifestation in *USP8*-Mutated Corticotropinoma May Be Mediated by Interactions Between WNT Signaling and SST Trafficking

**DOI:** 10.3390/ijms252312886

**Published:** 2024-11-29

**Authors:** Elena Nerubenko, Pavel Ryazanov, Natalia Kuritsyna, Artem Paltsev, Oksana Ivanova, Elena Grineva, Anna Kostareva, Renata Dmitrieva, Uliana Tsoy

**Affiliations:** Almazov National Medical Research Centre, 197341 Saint Petersburg, Russia; ryazanov_pa@almazovcentre.ru (P.R.); kuritsyna_nv@almazovcentre.ru (N.K.); paltsev_aa@almazovcentre.ru (A.P.); oksana.ivanova.al@gmail.com (O.I.); grineva_en@almazovcentre.ru (E.G.); kostareva_aa@almazovcentre.ru (A.K.); renata.i.dmitrieva@gmail.com (R.D.)

**Keywords:** Cushing’s disease, corticotropinomas, pituitary adenomas, PitNETs, *USP8* variants, somatostatin receptors, SST2, SST5, Wnt signaling

## Abstract

In the current work, we aimed to evaluate the association of clinical data of Cushing’s disease (CD) patients with *USP8* mutation status and to study USP8-related molecular mechanisms connected to the regulation of corticotropinoma growth and activity. 35 CD patients were enrolled; the sequencing of exon 14 in *USP8* revealed variants in eighteen adenomas, two of which were described for the first time in CD. *USP8* variants were more common in women (94% vs. 76%; *p* = 0.001), and microadenomas and tumor recurrence were prevalent in the *USP8*-mutant group (44% vs. 29%; *p* = 0.04 and 44% vs. 22%; *p* = 0.0015). Preoperative ACTH and serum cortisol did not differ in the *USP8*-WT and *USP8*-mutant patients. All *USP8*-mutant adenomas were SST5-positive, and 73% of them were double-positive (SST5+/SST2+). A total of 50% of *USP8*-WT adenomas were double-negative (SST5−/SST2−), and 40% of them were SST5-positive. Analysis of transcriptome was performed for nine *USP8*-mutant and six *USP8*-WT adenomas and revealed the that the bidirectional dysregulation of Wnt signaling, including both the agonist RSPO2 and antagonist SFRP1, in the *USP8*-mutant corticotropinomas was downregulated. These alterations may indicate the existence of regulatory connections between USP8 enzyme activity, Wnt signaling, EGFR signaling and somatostatin receptors’ trafficking, which can explain, at least in part, the clinical manifestations of CD in patients with corticotropinomas harboring *USP8* variants.

## 1. Introduction

Cushing’s disease (CD) is a severe endocrine disorder associated with increased morbidity and mortality due to cardiovascular and cerebrovascular complications, thromboembolism, diabetes, osteoporosis, fractures and infections [1]. The cause of CD is an adrenocorticotropic hormone (ACTH)-producing pituitary neuroendocrine tumor (PitNET), also named a corticotropinoma [2]. The prevalence of CD is considered about 40 cases per million people, and its incidence is 0.7 to 2.4 new cases per million per year [3,4,5].

Somatic mutations in *USP8* have been detected in a significant fraction of corticotropinomas [6,7,8,9]. It remains unknown how *USP8* mutations affect the regulation of tumor growth and the severity of disease. Some authors report the association of *USP8* with the severity of CD [10,11], while others report no association [7,12]. Normally, CD-associated *USP8* variants are gain-of-function mutations clustered in a hotspot region in exon 14 encoding the 14-3-3 binding motif (RSYSSP) [7,13].

The data on the association of *USP8* variants with clinical manifestations of CD and tumor growth are controversial [7,11,12,13,14]. Hayashi K. and coauthors [14] showed a significant decrease in ACTH secretion and tumor size in patients with *USP8* mutations, while others have found no differences in their plasma ACTH [7,12], morning serum cortisol [7,12,14], 24 h cortisol excretion [13], or maximum tumor size [12]. It has been reported that recurrences are more common among patients carrying the mutation [11].

Works studying the molecular mechanisms related to somatic mutations in *USP8* are limited. There is evidence that *USP8* mutations in the hotspot region of exon 14 result in the increased catalytic activity of USP8, leading to the deubiquitination of the epidermal growth factor receptor (EGFR) and the activation of EGFR recycling and EGFR-induced signaling essential for the production of proopiomelanocortin (POMC), which is a precursor of ACTH [13].

The expression of somatostatin receptors (SST) on cell membranes is important for tumors’ ability to achieve the binding of the somatostatin ligand, the activation of antiproliferative signal transduction and the inhibition of hormone secretion [15]. In the human pituitary gland, SST subtype 2 (SST2) and SST subtype 5 (SST5) are the most common [16]. The expression of SST in different PitNETs is various and depends on numerous factors. As for corticotropinomas, it is generally accepted that in CD the expression of SST2, but not of SST5, is downregulated by the high levels of cortisol [17].

The aim of this study was to evaluate clinical data of a cohort of CD patients with different *USP8* mutation statuses and to perform transcriptome analysis of tumor tissue to define USP8-related molecular mechanisms determining the biological features of corticotropinoma.

## 2. Results

### 2.1. Clinical Data

Thirty-five patients (twenty-nine women) with CD, with a median age of 38 years [31; 55] (18–67) were enrolled into this study. Clinical data for the whole cohort of patients are shown in Table S1. The histological examination of resected tissue confirmed pituitary adenomas, and immunohistochemistry staining (IHC) showed ACTH expression in all cases.

### 2.2. USP8 Mutations Spectrum in Patients with Cushing’s Disease

**Figure 1:**
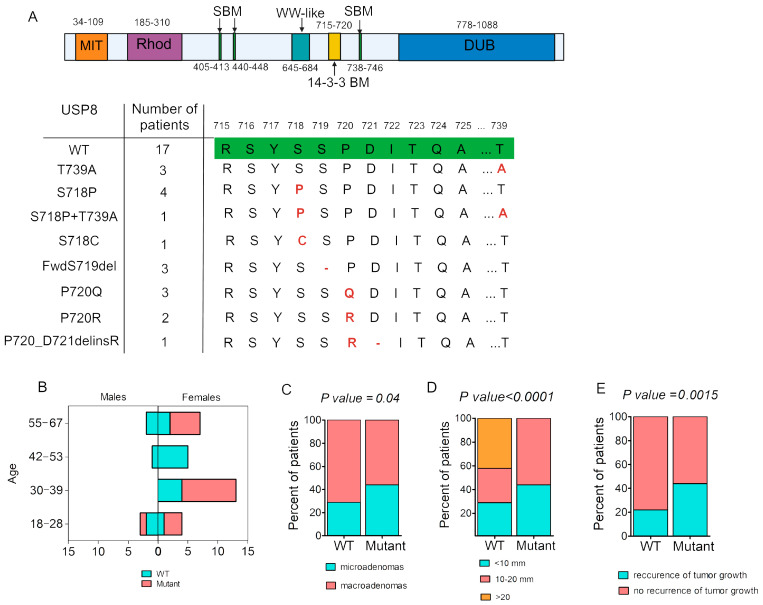
*USP8* mutation spectrum in our cohort of patients with Cushing’s disease and association of carrying *USP8* mutation with clinical data. (**A**) *USP8* structure: microtubule-interacting and trafficking domain (MIT), rhodanese-like domain (Rhod), SH3-binding motif (SBM), WW-like domain, 14-3-3-binding motif (14-3-3 BM, «hotspot» region), and deubiquitinase catalytic domain (DUB) that removes the conjugated ubiquitin molecules from the target proteins. Exon 14 sequencing region showing previously described variants located in «hotspot» region; identifying novel variant P720_D721delinsR as overlapping with «hotspot» region; and mutation T739A out of «hotspot» region, located in SBM3. Green indicates the wild-type sequence, and red highlights the mutation that replaces the wild-type sequence. (**B**) Histograms representing distribution of cases with *USP8*-WT and *USP8*-mutant among males and females in different age groups. (**C**) Prevalence of pituitary microadenomas and macroadenomas among *USP8*-mutant and *USP8*-WT corticotropinomas. Data were analyzed using Fisher’s exact test: microadenomas, *n*(Mutant) = 8(44%) and *n*(WT) = 5(29%); macroadenomas, *n*(Mutant) = 10(56%) and *n*(WT) = 12(71%). (**D**) Size of pituitary adenomas in groups with different *USP8* mutation statuses. Bar plot representing distribution of adenoma size in all patients. Data were analyzed using Chi-square test: *n*(Mutant, <10 mm) = 8(44%); *n*(WT, <10 mm) = 5(29%); n(Mutant, 10–20 mm) = 10(56%); *n*(WT, 10–20 mm) = 5(29%); and *n*(WT, >20 mm) = 7(42%). (**E**) Recurrence rate of tumor growth during follow-up period in USP8-mutant and USP8-WT groups. Data were analyzed using Fisher’s exact test: recurrence, *n*(Mutant) = 8(44%) and *n*(WT) = 2(22%); no recurrence, *n*(Mutant) = 10(56%) and *n*(WT) = 7(78%). WT—cases with absence of *USP8* mutation. Mutant—cases with presence of *USP8* mutation.

Fifty-one percent (18/35) of adenomas harbor *USP8* mutations. Seven different variants of the *USP8* gene were identified including five missense mutations and two deletions, as summarized in Figure 1A. The variants Fwd719del, P720R, S718P and P720Q have been described previously [18]. The variant T739A, located outside the «hotspot» region, was shown in CD patients for the first time in our recent work [19]; in one case this mutation was combined with the variant S718P. Another novel *USP8* variant, P720_D721delinsR, is located at the border of the «hotspot» region.

### 2.3. Associations Between Somatic USP8 Mutation and Clinical Parameters

The clinical and demographic parameters of patients with *USP8*-mutant and *USP8*-wild type (*USP8*-WT) corticotropinomas are presented in Table 1, Tables S1 and S2 and Figure 1B–E.

Variants in *USP8* were more common in women (94% vs. 76%; *p* = 0.001). Microadenomas prevailed in the *USP8*-mutant tumors (44% vs. 29%; *p* = 0.04) (Table 1, Figure 1C). Furthermore, all tumors ≥ 20 mm were *USP8*-WT tumors (Figure 1D). Plasma ACTH, morning serum cortisol and 24 h UFC levels did not differ between the *USP8*-mutant and *USP8*-WT groups. (shown in Table 1). This being considered, macroadenomas were later excluded from analysis (shown in Table S2). It is noteworthy that the size of the microadenomas did not differ between the *USP8*-mutant and *USP8*-WT tumors (shown in Table S2). Despite the similar levels of ACTH and cortisol in the patients with *USP8*-mutant and *USP8*-WT corticotropinomas, the mortality and necessity of BLAE were perceived in the WT group (Table S1).

The long-term follow-up period was similar for the *USP8*-mutant and *USP8*-WT cases (2 years, [1; 3]; (1–9) vs. 2 years, [1; 3]; (1–10) *p* = 0.94). Of the 18 patients with *USP8*-mutant pituitary adenomas, hypercortisolism remission was proved in 10 cases (55%). Among the nine patients with *USP8*-WT corticotropinomas, the remission of hypercortisolism was confirmed in six cases (60%).

Of the eighteen patients with *USP8*-mutant corticotropinomas, the recurrence of pituitary adenoma growth was detected in eight (44%) cases, and among nine patients with *USP8*-WT tumors, the continued growth of pituitary adenomas was confirmed in two (22%) cases. In the patient who underwent BLAE 1.5 year after TSS, adenoma growth was not detected during the next 3 years of follow up. Two patients with Nelson’s syndrome were excluded from this analysis. Thus, the frequency of hypercortisolism remission did not differ between the *USP8*-mutant and *USP8*-WT patients (*p* = 0.57) but the recurrence of pituitary adenoma growth was more common (*p* = 0.0015) in corticotropinomas with *USP8* variants (shown in Figure 1E).

### 2.4. SST2/SST5 Expression and USP8 Mutation Status

**Figure 2:**
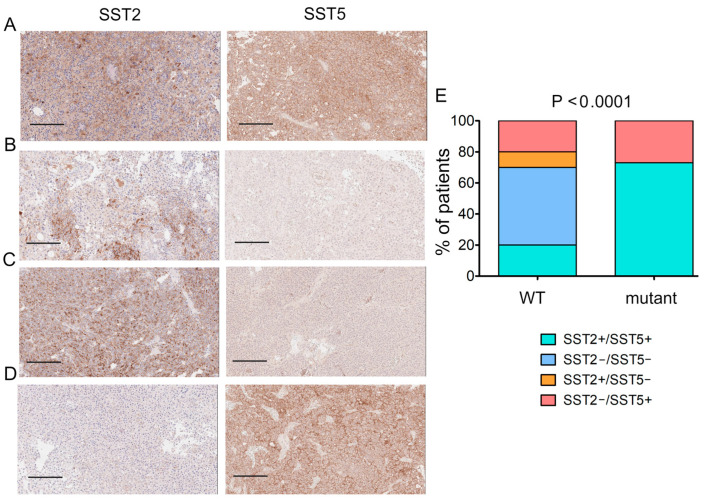
IHC expression of SST2/5 between *USP8*-mutant and *USP8*-WT in CD patients. Data were analyzed using Chi-square test. (**A**–**D**) Examples of different patterns of SST2/SST5 expression: (**A**) patient P1 with double-positive expression of SST2+/SST5+; (**B**) patient P21 with double-negative expression of SST2−/SST5−; (**C**) patient P5 with positive expression of SST2+ and negative expression of SST5−; (**D**) patient P20 with negative expression of SST2− and with positive expression of SST5+; (**E**) percentage of patients with each kind of SST2/5 expression in WT and mutant tumors. Scale bar = 200 μm. IHC—immunohistochemical staining.

Staining for SST2 and SST5 receptors was performed in 23 corticotropinomas, 12 in *USP8*-mutant and 11 in *USP8*-WT samples. The expression of both types of SST was calculated for 21 corticotropinomas (Figure 2); in two cases, only SST2 or SST5 receptors were stained due to the limited amount of adenoma tissue (Table S1).

All mutant adenomas were SST5+, and most of them (8/11) were double-positive (SST2+/SST5+); only four out of the ten *USP8*-WT adenomas were SST5+, and two out of the ten were SST2+/SST5+ (Figure 2E). Out of the six SST5-negative *USP8*-WT adenomas, five were double-negative (SST2−/SST5−) and one was SST2+/SST5−.

### 2.5. Transcriptome Analysis of Pituitary Adenomas Harboring USP8-Mutant or USP8-WT in Patients with Cushing’s Disease

To uncover the possible molecular mechanisms that might be responsible for the differences between the *USP8*-mutant and *USP8*-WT adenomas, we performed transcriptome analysis.

**Figure 3:**
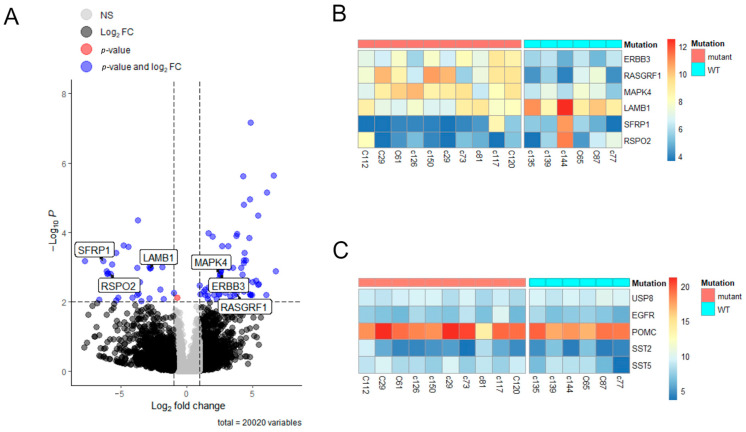
Distinct gene expression profile between *USP8*-mutant and *USP8*-WT in ACTH-secreting pituitary adenomas. (**A**) Volcano plot of differently expressed genes between *USP8*-mutant and *USP8*-WT. DEGs upregulated (right side) and downregulated (left side) in *USP8*-mutant. Blue dots are DEGs. *Y* axis is significant differences in gene expression between groups with FDR < 0.01. *X* axis is Log_2_ fold changes in gene expression with cutoff |Log_2_FC| > 1. (**B**) Heatmap of specific DEGs’ expression. (**C**) Heatmap of *USP8*, *EGFR*, *POMC*, *SST2* and *SST5* expression which are not related to DEGs between *USP8*-mutant and *USP8*-WT.

Though the principal component analysis (PCA) showed no clear separation between the groups (Figure S1A), the analysis of the differentially expressed genes (DEGs) revealed 52 downregulated genes and 80 upregulated genes in samples from the *USP8*-mutant tumors (Figure S1B; the whole list of DEGs associated with *USP8* mutations in adenomas is given in Supplementary Table S3). Also, the results of the analysis are visualized on a volcano plot (Figure 3A).

To reveal the biological functions and regulatory mechanisms that could be associated with the identified DEGs, we performed pathway enrichment analysis and found a few gene signatures associated with important molecular mechanisms that may be involved in the regulation of tumor growth. Thus, in *USP8*-mutant samples, we found significant alterations in the expression of genes associated with the regulation of MAPK family signaling cascades (*ERBB3*, *RASGRF1*, *MAPK4*), regulation of epithelial cell proliferation (*SFRP1*, *LAMB1*) and regulation of the Wnt signaling pathway (*SFRP1*, *RSPO2*). All these genes are shown on the volcano plot (Figure 3A) and visualized on a heatmap in Figure 3B; we found no difference in the expression of *USP8*, *EGFR*, *POMC*, *SST2* and *SST5* (Figure 3C). Interestingly, all these genes are related to tumor biology in different ways, and most of them were shown to be involved in USP8-mediated processes.

## 3. Discussion

In our work, we aimed to evaluate the clinical significance of somatic *USP8* variants in corticotropinomas derived from patients with CD and to clarify the molecular mechanisms of the found effects. In our cohort of 35 patients with CD, we detected *USP8* variants within or close to the 14-3-3 downregulatory site in 18. The main clinical findings in the patients with corticotropinomas harboring *USP8* variants were the prevalence of microadenomas, more frequent recurrence after successful surgery and the prevailing of SST5 and SST2 receptors’ expression. The analysis of transcriptome showed the possible regulatory connections between the *USP8* mutations and clinical features.

When analyzing our own data and data from other researchers, we noticed the duality of *USP8*-mutated corticotropinomas’ clinical manifestations. As it has been described before, in corticotropinomas harboring *USP8* variants, the increased deubiquitination of EGFR results in decreased EGFR lysosomal degradation and the activation of EGFR signaling, which contributes to oncogenic transformation in aggressive tumors [13,20,21]. Indeed, the more frequent recurrence of *USP8*-mutated corticotropinomas was shown after successful neurosurgery in our study and by others [22,23,24]. At the same time, in our cohort, microadenomas prevailed in the *USP8*-mutant group. Furthermore, the maximum diameter of all *USP8*-mutant corticotropinomas did not exceed 2 cm, while about 40% of *USP8*-WT tumors were >2 cm. In vitro studies have revealed that in corticotropinomas harboring *USP8* variants the activation of EGFR signaling enhances proopiomelanocortin (POMC) transcription and ACTH hyperproduction [13,14,20], supporting the hypothesis that *USP8*-mutant corticotropinomas more actively produce ACTH. However, in our study, we did not show differences in the morning plasma ACTH and morning serum cortisol levels in patients with *USP8*-mutant and *USP8*-WT tumors. The same data were found after the macroadenomas were excluded to offset the effect of tumor size (Table S2). The association of more severe hypercortisolism with *USP8* variants was also not found; all patients who died, and those who underwent BLAE, harbored a *USP8*-WT tumor.

Thus, we assumed the existence of another USP8-dependent mechanism, opposing the canonical effects on EGFR signaling and providing the dualistic effects of USP8 activation in corticotropinomas harboring *USP8* variants. Our results on the expression of SST5 and SST2 in corticotropinomas support this hypothesis. In this work, we detected SST5 expression in all *USP8*-mutant tumors, while only a small fraction of *USP8*-WT was SST5 positive, which fits the data obtained by others well [14,25,26,27,28]. An unexpected finding was positive SST2 expression in the majority of *USP8*-mutant corticotropinomas (in eight of eleven cases) and in three of the ten *USP8*-WT tumors. Most previous studies have shown low or no expression of both SST2 mRNA and protein in ACTH-secreting PitNETs [14,26,28,29,30,31,32]. This may be explained by the data obtained in AtT-20 pituitary adenoma cell lines, which have demonstrated the suppression of SST2 expression in corticotropinomas under glucocorticoid excess [29]. Somatostatin receptors, including SST5 and SST2, are known to mediate the action of somatostatin, which downregulates different hormones’ production and suppresses cell proliferation by potentiating cell cycle arrest and apoptosis [17]. Thus, considering our clinical data and data on the predominant expression of SST5 and SST2 in *USP8*-mutant tumors, we hypothesize that *USP8* mutation may result in multidirectional alterations in the regulation of cell growth and proliferation and hormonal production in corticotropinomas. The duality of changes induced by *USP8* mutation in corticotropinomas and alterations in the balance between proliferative and antiproliferative effects in each individual case can explain the inconsistency of clinical data obtained by researchers from CD patients with corticotropinomas with different *USP8* mutation statuses [7,13,14,20,22,23,33,34].

Though the analysis of transcriptome did not reveal differences in *SST5/SST2* between the *USP8*-mutant and *USP8*-WT tumors, there were differences in the SST5/SST2 proteins’ presence on the tumors’ cell membranes, possibly due to alterations in mechanisms that regulate receptors’ internalization, recycling and lysosomal degradation [35,36], which can be mediated by both USP8 activity and WNT signaling.

Although there are data showing that SST5 is not ubiquitinated after treatment with an SST5-specific ligand, and there is no direct effect of USP8 on SST5 at least in in vitro AtT-20 mouse pituitary gland cells [37], data exist that show that Dvl1 (the WNT pathway’s protein) may regulate SST2 receptor ubiquitination and degradation in a ligand-independent manner through interaction with the E3 ubiquitin ligases ZNRF3/RNF43 [38]. We speculate that similar mechanisms may also affect SST5, contributing to the observed receptor expression patterns. WNT signaling, as well as EGFR signaling, is known to be activated in corticotropinomas [39,40]. Furthermore, the role of USP8 activity in the modulation of Wnt signaling via influencing Fzd receptors, whose activation is essential for Wnt signaling [41], has been described [42,43,44]. It has been shown that the gain and loss of USP8 function has led to the up- and downregulation, respectively, of canonical Wnt signaling due to an alteration in the balance between the ubiquitylation and deubiquitylation of Fzd receptors [42,43].

In this work, we have shown the significant downregulation of *RSPO2* (agonist) and *SFRP1* (antagonist) in WNT signaling in *USP8*-mutant corticotropinomas, which supports our hypothesis on the role of *USP8*-mutation-altered WNT signaling regulation in these tumors. SFRP1- and RSPO2-dependent mechanisms are known to be among those controlling Fzd cell membrane expression [41,45]. RSPO2 negatively controls the activity of the E3 ubiquitin ligases ZNRF3/RNF43 for preventing the ubiquitination of Fzd and for the activation of WNT signaling [46], while SFRP1 inhibits Wnt pathway activation through preventing WNT ligand coupling with Fzd [45]. Interestingly, EGFR cell membrane expression is also controlled by the E3 ligases ZNRF3/RNF43 in the same way as Fzd [47].

We speculate that the downregulation of *RSPO2* in *USP8*-mutant corticotropinomas may result in the activation of the E3 ubiquitin ligases ZNRF3/RNF43 and, as a consequence, the increased recruitment of Dvl1 for Fzd degradation. In this case, Dvl1-mediated SST receptor ubiquitination and degradation may be depleted in *USP8*-mutant corticotropinomas.

To summarize, we assume that the existence of mechanistical connections between USP8 enzyme activity, Wnt signaling, EGFR signaling and somatostatin (SST) receptors’ trafficking can explain, at least in part, the clinical alterations found in CD patients harboring corticotropinomas with *USP8* variants. Importantly, all the parts of the complicated network involved in the control of the Wnt signaling pathway are subjects for feedback loop regulations [41]; therefore, disturbances in any part of this regulatory system would result in multidirectional biological and clinical outcomes.

This study has some limitations; one of them is the small size of the group. The possibility to collect a larger sample in one center is hindered by CD’s peculiarities. This rare pathology is mainly caused by corticotropinomas of small sizes, and sufficient material for histological, immunohistochemical and genetic studies is not always available. Genetic testing was performed only when pathology examination, including immunohistochemistry, had confirmed the diagnosis. We did not test the samples for the presence of corticotropinoma cells before the genetic testing, and this may be another limitation of our study. But the high prevalence of *USP8* variants among the microadenomas supports our proposal that the pathology confirmation was sufficient. The results of the transcriptomic analysis and our hypothesis on the interaction between different pathways in *USP8*-mutated corticotropinomas need to be confirmed in additional experimental works with in vitro cellular models, and this is in the scope of our future investigations.

## 4. Materials and Methods

### 4.1. Study Population of Patients with Cushing’s Disease

This study was conducted in accordance with the Declaration of Helsinki and approved by the Institutional Review Board (or Ethics Committee) of Almazov National Medical Research Centre (protocol code, 193-4, date of approval, 11 April 2016). All patients signed an institutional review board-approved statement of informed consent. The study population comprised 35 CD patients who underwent transsphenoidal surgery (TSS) from 2012 to 2021 in the Federal Almazov National Medical Research Centre (Table S1). CD was diagnosed according to the current guidelines [48,49]. Endogenous hypercortisolism was confirmed based on midnight serum or salivary cortisol levels, elevated 24 h urinary free cortisol (UFC) levels and a lack of cortisol suppression after a low dexamethasone suppression test (LDST) [48,49]. ACTH-dependent syndrome was diagnosed if the ACTH plasma level was >2 pmol/L [50]. Dynamic pituitary magnetic resonance imaging (MRI) was performed for corticotropinoma visualization. When the MRI pituitary adenoma size was <8 mm, bilateral cavernous and inferior petrosal sinuses sampling was performed as described in [51,52,53].

Twenty-seven patients underwent a primary operation, and six repeated the operation due to adenoma relapse. In two patients, surgery was performed for Nelson’s syndrome (P26, P35—patients’ IDs, shown in Table S1). Histological examination of resected tissue confirmed pituitary adenomas, and immunohistochemistry staining (IHC) showed ACTH expression.

In the late postoperative period (2–3 weeks after TSS), three patients died: one (c77—patient ID, shown in Table S1) died due to a massive pulmonary embolism and two died due to the fulminant course of acute hepatitis B (P24—patient ID, shown in Table S1) and bacterial pneumonia (P21—patient ID, shown in Table S1).

Five patients underwent bilateral adrenalectomy (BLAE), including two with Nelson’s syndrome. One patient (c144—patient ID, shown in Table S1) with an invasive pituitary macroadenoma underwent BLAE a month before TSS because of the severe hypercortisolism complicated by persistent infection that interfered with pituitary surgery.

In two cases, BLAE was performed after TSS; in one (P24—patient ID, shown in Table S1), it was performed two weeks after non-radical TSS due to severe hypercortisolism that was resistant to medical therapy, and in the other (PK—patient ID, shown in Table S1), it was performed 1.5 years after TSS because of the CD’s persistence despite medical treatment and an inability to perform repeated TSS or radiosurgery due to unfavorable MRI data.

The late postoperative results of the TSS were evaluated for 31 out of the 35 patients. In twenty-six patients, adrenal insufficiency developed after TSS, and in five, hypercortisolism persisted. Long-term results were analyzed for patients with a follow-up period one year or more; the median of follow-up period was two years [1; 4], (1–9). In patients with pituitary adenoma regrowth, the time of tumor relapse was considered the end of follow-up period.

Long-term follow-up data were obtained for twenty-nine out of the thirty-five patients; among the other six, three patients died and contact was lost with the three others. Of the two patients with Nelson’s syndrome, adenoma growth was confirmed for one. The recurrence of pituitary adenoma was detected by MRI in 10 cases, in 17 cases, including a patient who underwent BLAE 1.5 years after TSS, pituitary adenoma relapse was absent. CD remission was observed in 16 cases, in 11 patients, hypercortisolism was confirmed. Remission was diagnosed if the need for glucocorticoid replacement therapy persisted or in the case of a combination of the following criteria: the normalization of 24 h UFC level, the normalization of midnight salivary and/or midnight serum cortisol levels and serum cortisol in LDST < 50 nmol/L.

#### 4.1.1. Hormone Measurements

Cortisol (serum, salivary, urinary) and ACTH (serum) were measured using enzyme immunoassays Cobas E411 (Roche Diagnostics, Indianapolis, IN, USA).

#### 4.1.2. Pituitary Magnetic Resonance Imaging

Dynamic pituitary magnetic resonance imaging (MRI) was performed after the intravenous administration of gadolinium-111In-diethylenetriamine-pentacetic acid. Magnetom Trio A Tim 3.0 T (Siemens Healthineers, Erlangen, Germany), with a detection limit of 2 mm, was used. A macroadenoma was defined as a pituitary tumor with a diameter ≥ 1 cm, and microadenomas were defined as those with a diameter < 1 cm.

#### 4.1.3. Immunnohistochemical (IHC) Staining

Paraffin-embedded fixed material was first deparaffinized/rehydrated using Xylol and a descending series of ethanol solutions. Then, the sections were pre-treated with 3% hydrogen peroxide (room temperature, 5 min) in order to inactivate endogenous peroxidase. Then, the sections were washed in distilled water. An antigen retrieval procedure was carried out in Tris-EDTA pH 9.0 for ACTH and SSTR5 and pH 6.0 for SSTR2 (Dako (Agilent Technologies), Glostrup, Denmark) at 95–98 °C for 35 min. Then, the material was cooled at room temperature. Subsequently, the sections were washed in Tris-buffered saline using Tween 20 two times for 5 min (Dako (Agilent Technologies), Glostrup, Denmark). The mounted sections were circumscribed with a hydrophobic pen Elite PAP Pen (Diagnostic BioSystems, Pleasanton, CA, USA). Immunolabeling was performed with the following antibodies: a mouse monoclonal antibody against ACTH at a dilution of 1:400 clone AH26 (Diagnostic BioSystems, Pleasanton, CA, USA), a rabbit monoclonal antibody SSTR2 at a dilution of 1:25 (Clone UMB-1, Epitomics, Inc., Burlingame, CA, USA) and a rabbit monoclonal antibody SSTR5 at a dilution of 1:100 (Clone UMB-4, Epitomics, Inc., Burlingame, CA, USA). After incubation with primary antibodies, the sections were washed two times in Tris-buffered saline using Tween 20. For the detection of primary antibodies, we used the Real EnVision Detection System, Peroxidase/DAB, Rabbit/Mouse kit (Dako (Agilent Technologies), Glostrup, Denmark). Incubation with the primary and secondary antibodies was performed for 30 min at room temperature. After washing in distilled water, the sections were counterstained with hematoxylin (2 min) and dehydrated and then coverslips were mounted using permanent mounting medium Polystyrol, BioMount (Bio-Optica, Milan, Italy). To quantify the SST staining, only tumor cell membrane expression was taken in account. The percentage of tumor cells with membrane expression (positive cells) was calculated as the mean from data collected after positive cells were counted in ten fields of view at a magnification of 200x. The staining results were scored as “+++” (if >50% cells showed SST expression), “++” (if ≥30–50% cells showed SST expression), “+” (if ≥10–30% cells showed SST expression) or “-” (if <10% cells showed SST expression). For the purposes of binary analysis, cases that scored “-” or “+” were considered negative, while those that scored “++” and “+++” were considered positive.

#### 4.1.4. Statistical Analysis

The results were presented as the median (Me), interquartile range [25%; 75%] and minimal and maximal values (min.–max.). The Mann–Whitney test was used for quantitative variables and Fisher’s exact or the Chi-square (for contingency table more than 2 × 2) tests were used for qualitative variables. Statistical significance was accepted for *p* < 0.05.

### 4.2. DNA/RNA Purification from Tumor Samples

The tumor samples were collected during TSS. A total of 19 samples were immediately snap-frozen in liquid nitrogen and stored at −80 °C. A total of 16 samples were stored as paraffin-embedded samples.

The Trizol (Thermo Fisher Scientific, Waltham, MA, USA) method of DNA/RNA extraction was used for the snap-frozen tissue samples according to manufacturer’s manuals. The process typically involves the following: The tissue is homogenized in Trizol reagent to break down cellular structures and release nucleic acids. Chloroform is added to separate the phases, allowing RNA to remain in the aqueous phase, while DNA and proteins separate into the organic and interphase layers, respectively. The aqueous phase, containing RNA, is then further purified, while the other layers are stored at −80 °C before further processing. During the Trizol DNA guide extraction, we added an additional step with 500 μL of BEB (back extraction buffer; 4 M guanidine thiocyanate, 50 mM sodium citrate and 1M Tris base) to maximize the DNA recovery. After adding the BEB, 10 min of incubation followed. Centrifugation at 12,000× *g* and 4 °C for 15 min followed, with the aqueous phase collected in a new tube, and was followed by further steps (precipitation with isopropanol, washing and elution) according to the Trizol user guide.

DNA from the paraffin-embedded tissue samples was extracted using the QIAamp DNA FFPE Kit (QIAGEN, Germantown, MD, USA) according to manufacturer’s manuals. The kit is specifically designed for the recovery of high-quality DNA from FFPE tissue, where DNA may be cross-linked or fragmented due to formalin fixation. The extraction process involves the following: deparaffinizing the tissue by using solvent to remove the paraffin wax; digesting the tissue with proteinase K to break down proteins and facilitate the release of DNA; using silica-based membrane technology to capture the DNA; and, to follow, washing and elution to recover purified DNA.

The DNA and RNA samples were carefully stored at −80 °C. The concentration and integrity of the extracted DNA were assessed using spectrophotometric measurements (using a NanoDrop) and gel electrophoresis to ensure that the nucleic acids were of a sufficient quality for downstream molecular analyses. For the RNA samples, quality control was performed with Bioanalyzer (Agilent Technologies, Santa Clara, CA, USA). Integrity was assessed by evaluating the RNA’s RIN (RNA integrity number).

### 4.3. Sanger Sequencing of USP8

Sanger sequencing of the *USP8*’s exon 14 was performed using the BigDye terminator Sequencing Kit (Thermo Fisher Scientific (Applied Biosystems), Foster City, CA, USA) and an ABI PRISM 3100 Genetic Analyzer (Thermo Fisher Scientific (Applied Biosystems), Foster City, CA, USA). The primers were designed using the NCBI Primer Blast forward, 5′-CCCAATCACTGGAACCTTTCG-3′, and reverse, 5′-CCAACTCCCTGACACTAACATAC-3′. Analysis of chromatograms was carried out in the Geneious Prime suite v2024.0.7 (Biomatters, Auckland, New Zealand) with the following NCBI reference sequence: NC_000015.10 Homo sapiens chromosome 15, GRCh38.p14 Primary Assembly (Table S4).

### 4.4. Transcriptome Sequencing and Analysis

Libraries for RNA sequencing were synthesized using the TruSeq Stranded mRNA kit (Illumina Inc., San Diego, CA, USA) and quality control was performed using the 4150 TapeStation system (Agilent, Santa Clara, CA, USA) using High Sensitivity DNA ScreenTape Analysis. Sequencing was performed using Illumina NextSeq 2000 with a P2 reagent cartridge and 100 cycles (2 × 50 bp). Reads were aligned to hg38 with gencode.v41.annotation.gtf using STAR v2.6.1a (Developed by Alexander Dobin and collaborators (University of NC at Chapel Hill), Chapel Hill, NC, USA). Mapped reads were counted with the featureCounts program v1.6.2 (Wei Shi and colleagues at the Department of Biomedical Informatics, Harvard Medical School, Boston, MA, USA). R v4.2.2 (developed by Ross Ihaka and Robert Gentleman, university of Auckland, New Zealand) was used for next data processing. The DESeq2 v1.38.0 (Developed by Michael Love, Rafael Irizarry, and the Bioconductor project, Boston, MA, USA) R package was used for analyzing the differential expression of genes. The genes’ *p* values were adjusted using the Benjamini–Hochberg procedure; only genes filtered with an FDR < 0.01 and |Log_2_FC| > 1 were considered differentially expressed genes (DEGs). The Enrichr web annotation tool (The Ma’ayan Lab, Icahn School of Medicine at Mount Sinai, New York City, NY, USA, https://maayanlab.cloud/Enrichr/, accessed on 21 October 2024) was used [54] for the identification of biological pathways related to the DEGs with *p* cutoff = 0.05.

## 5. Conclusions

In corticotropinomas harboring *USP8* variants, we showed the activation of different intracellular multidirectional processes whose cross-talks may lead to a variety of manifestations of CD. We assume that *USP8* mutation-associated dysregulations of Wnt signaling may result in alterations in SST2/5 receptors’ trafficking causing alterations in molecular mechanisms that control tumor growth and progression. The impact of *USP8* mutation on the relationship between Wnt signaling, EGFR signaling and SST trafficking in corticotropinomas needs further investigations.

## Figures and Tables

**Table 1:** Clinical data and comparison of patients with USP8-mutant and USP8-WT corticotropinomas.

Clinical Data	*USP8*-Mutant	*USP8*-WT	*p*
Sex, m/f, %	6%/94%	24%/76%	*p* = 0.001
Age, years Me [25%; 75%] (min.–max.)	36.5 [29; 56.5] (18–67)	45 [34; 54] (21–60)	*p* = 0.43
Microadenomas/macroadenomas, %	44%/56%	29%/71%	*p* = 0.04
Maximum adenoma size, mm Me [25%; 75%] (min.–max.)	11.25 [8; 15] (3–18)	12 [6; 30] (4–49)	*p* = 0.21
Adenoma volume, mL Me [25%; 75%] (min.–max.)	0.27 [0.1; 0.43] (0.003–1.56)	0.37 [0.043; 6.23] (0.01–42.34)	*p* = 0.25
Morning plasma ACTH *, pg/mL Me [25%; 75%] (min.–max.)	61.57 [50.56; 81.76] (23.54–128.3)	82.99 [45.67; 161.2] (15.34–645.6)	*p* = 0.39
Morning serum cortisol *, nmol/L Me [25%; 75%] (min.–max.)	735 [588.2; 837.3] (448–1094)	638.85 [430.1; 831.6] (261–1690)	*p* = 0.51
24 h UFC *, nmol/24 h Me [25%; 75%] (min.–max.)	492.5 [271.3–696.6] (137.88–1752)	570 [195.8; 861.8] (56.8–3392)	*p* = 0.93
Ki67, %	1 [0.4; 3.27] (0.1–5.3)	1.47 [0.53; 3.64] (0.1–17.0)	*p* = 0.6

24 h UFC—24-h urinary free cortisol; ACTH—adrenocorticotropic hormone; Me—median; * patients with Nelson’s syndrome were excluded.

## Data Availability

The RNA-seq data are available from the GEO public database with the accession number GSE263206.

## Supplementary Materials

The following supporting information can be downloaded at: https://www.mdpi.com/article/10.3390/ijms252312886/s1.

## References

[1] Dekkers O.M., Biermasz N.R., Pereira A.M., Roelfsema F., Van Aken M.O., Voormolen J.H.C., Romijn J.A. (2007). Mortality in Patients Treated for Cushing’s Disease Is Increased, Compared with Patients Treated for Nonfunctioning Pituitary Macroadenoma. J. Clin. Endocrinol. Metab..

[2] Asa S.L., Mete O., Perry A., Osamura R.Y. (2022). Overview of the 2022 WHO Classification of Pituitary Tumors. Endocr. Pathol..

[3] Lindholm J., Juul S., Jørgensen J.O.L., Astrup J., Bjerre P., Feldt-Rasmussen U., Hagen C., Jørgensen J., Kosteljanetz M., Kristensen L.Ø. (2001). Incidence and Late Prognosis of Cushing’s Syndrome: A Population-Based Study. J. Clin. Endocrinol. Metab..

[4] Etxabe J., Vazquez J.A. (1994). Morbidity and Mortality in Cushing’s Disease: An Epidemiological Approach. Clin. Endocrinol..

[5] Ragnarsson O., Olsson D.S., Chantzichristos D., Papakokkinou E., Dahlqvist P., Segerstedt E., Olsson T., Petersson M., Berinder K., Bensing S. (2019). The Incidence of Cushing’s Disease: A Nationwide Swedish Study. Pituitary.

[6] Huang C., Shi Y., Zhao Y. (2015). USP8 Mutation in Cushing’s Disease. Oncotarget.

[7] Perez-Rivas L.G., Theodoropoulou M., Ferraù F., Nusser C., Kawaguchi K., Stratakis C.A., Rueda Faucz F., Wildemberg L.E., Assié G., Beschorner R. (2015). The Gene of the Ubiquitin-Specific Protease 8 Is Frequently Mutated in Adenomas Causing Cushing’s Disease. J. Clin. Endocrinol. Metab..

[8] Wanichi I.Q., de Paula Mariani B.M., Frassetto F.P., Siqueira S.A.C., de Castro Musolino N.R., Cunha-Neto M.B.C., Ochman G., Cescato V.A.S., Machado M.C., Trarbach E.B. (2019). Cushing’s Disease Due to Somatic USP8 Mutations: A Systematic Review and Meta-Analysis. Pituitary.

[9] Albani A., Theodoropoulou M., Reincke M. (2018). Genetics of Cushing’s Disease. Clin. Endocrinol..

[10] Sesta A., Cassarino M.F., Terreni M., Ambrogio A.G., Libera L., Bardelli D., Lasio G., Losa M., Pecori Giraldi F. (2020). Ubiquitin-Specific Protease 8 Mutant Corticotrope Adenomas Present Unique Secretory and Molecular Features and Shed Light on the Role of Ubiquitylation on ACTH Processing. Neuroendocrinology.

[11] Treppiedi D., Barbieri A.M., Di Muro G., Marra G., Mangili F., Catalano R., Esposito E., Ferrante E., Serban A.L., Locatelli M. (2021). Genetic Profiling of a Cohort of Italian Patients with Acth-Secreting Pituitary Tumors and Characterization of a Novel Usp8 Gene Variant. Cancers.

[12] Bujko M., Kober P., Boresowicz J., Rusetska N., Zeber-Lubecka N., Paziewska A., Pekul M., Zielinski G., Styk A., Kunicki J. (2021). Differential MicroRNA Expression in USP8-Mutated and Wild-Type Corticotroph Pituitary Tumors Reflect the Difference in Protein Ubiquitination Processes. J. Clin. Med..

[13] Reincke M., Sbiera S., Hayakawa A., Theodoropoulou M., Osswald A., Beuschlein F., Meitinger T., Mizuno-Yamasaki E., Kawaguchi K., Saeki Y. (2015). Mutations in the Deubiquitinase Gene USP8 Cause Cushing’s Disease. Nat. Genet..

[14] Hayashi K., Inoshita N., Kawaguchi K., Ardisasmita A.I., Suzuki H., Fukuhara N., Okada M., Nishioka H., Takeuchi Y., Komada M. (2016). The USP8 Mutational Status May Predict Drug Susceptibility in Corticotroph Adenomas of Cushing’s Disease. Eur. J. Endocrinol..

[15] Theodoropoulou M., Stalla G.K. (2013). Somatostatin Receptors: From Signaling to Clinical Practice. Front. Neuroendocrinol..

[16] Ben-Shlomo A., Melmed Shlomo S. (2010). Pituitary Somatostatin Receptor Signaling. Trends Endocrinol. Metab..

[17] Cuevas-Ramos D., Fleseriu M. (2014). Somatostatin Receptor Ligands and Resistance to Treatment in Pituitary Adenomas. J. Mol. Endocrinol..

[18] Treppiedi D., Marra G., Di Muro G., Esposito E., Barbieri A.M., Catalano R., Mangili F., Bravi F., Locatelli M., Lania A.G. (2022). P720R USP8 Mutation Is Associated with a Better Responsiveness to Pasireotide in ACTH-Secreting PitNETs. Cancers.

[19] Petukhova N., Poluzerova A., Bug D., Nerubenko E., Kostareva A., Tsoy U., Dmitrieva R. (2024). USP8 Mutations Associated with Cushing’s Disease Alter Protein Structure Dynamics. Int. J. Mol. Sci..

[20] Ma Z.Y., Song Z.J., Chen J.H., Wang Y.F., Li S.Q., Zhou L.F., Mao Y., Li Y.M., Hu R.G., Zhang Z.Y. (2015). Recurrent Gain-of-Function USP8 Mutations in Cushing’s Disease. Cell Res..

[21] Mizuno E., Iura T., Mukai A., Yoshimori T., Kitamura N., Komada M. (2005). Regulation of Epidermal Growth Factor Receptor Down-Regulation by UBPY-Mediated Deubiquitination at Endosomes. Mol. Biol. Cell.

[22] Albani A., Pérez-Rivas L.G., Dimopoulou C., Zopp S., Colón-Bolea P., Roeber S., Honegger J., Flitsch J., Rachinger W., Buchfelder M. (2018). The USP8 Mutational Status May Predict Long-Term Remission in Patients with Cushing’s Disease. Clin. Endocrinol..

[23] Losa M., Mortini P., Pagnano A., Detomas M., Cassarino M.F., Pecori Giraldi F. (2019). Clinical Characteristics and Surgical Outcome in USP8-Mutated Human Adrenocorticotropic Hormone-Secreting Pituitary Adenomas. Endocrine.

[24] Faucz F.R., Tirosh A., Tatsi C., Berthon A., Hernández-Ramírez L.C., Settas N., Angelousi A., Correa R., Papadakis G.Z., Chittiboina P. (2017). Somatic USP8 Gene Mutations Are a Common Cause of Pediatric Cushing Disease. J. Clin. Endocrinol. Metab..

[25] Castellnou S., Vasiljevic A., Lapras V., Raverot V., Alix E., Borson-Chazot F., Jouanneau E., Raverot G., Lasolle H. (2020). SST5 Expression and USP8 Mutation in Functioning and Silent Corticotroph Pituitary Tumors. Endocr. Connect..

[26] Chinezu L., Vasiljevic A., Jouanneau E., François P., Borda A., Trouillas J., Raverot G. (2014). Expression of Somatostatin Receptors, SSTR2A and SSTR5, in 108 Endocrine Pituitary Tumors Using Immunohistochemical Detection with New Specific Monoclonal Antibodies. Hum. Pathol..

[27] Fuchs T.L., Sioson L., Sheen A., Clarkson A., Gill A.J. (2018). Immunohistochemical Expression of Somatostatin Receptors SSTR2A and SSTR5 in 299 Pituitary Adenomas. Pathology.

[28] Tateno T., Kato M., Tani Y., Oyama K., Yamada S., Hirata Y. (2009). Differential Expression of Somatostatin and Dopamine Receptor Subtype Genes in Adrenocorticotropin (ACTH)-Secreting Pituitary Tumors and Silent Corticotroph Adenomas. Endocr. J..

[29] van der Hoek J., Lamberts S.W.J., Hofland L.J. (2004). The Role of Somatostatin Analogs in Cushing’s Disease. Pituitary.

[30] Hofland L.J., van der Hoek J., Feelders R., van Aken M.O., van Koetsveld P.M., Waaijers M., Sprij-Mooij D., Bruns C., Weckbecker G., de Herder W.W. (2005). The Multi-Ligand Somatostatin Analogue SOM230 Inhibits ACTH Secretion by Cultured Human Corticotroph Adenomas via Somatostatin Receptor Type 5. Eur. J. Endocrinol..

[31] Lupp A., Hunder A., Petrich A., Nagel F., Doll C., Schulz S. (2011). Reassessment of Sst(5) Somatostatin Receptor Expression in Normal and Neoplastic Human Tissues Using the Novel Rabbit Monoclonal Antibody UMB-4. Neuroendocrinology.

[32] Hassaneen W., Cahill D.P., Fuller G.N., Levine N.B. (2010). Immunohistochemical Detection of Somatostatin Receptor Subtype 5 (SSTR-5) in Cushing Adenoma. J. Neurooncol..

[33] Martins C.S., Camargo R.C., Coeli-Lacchini F.B., Saggioro F.P., Moreira A.C., De Castro M. (2020). USP8 Mutations and Cell Cycle Regulation in Corticotroph Adenomas. Horm. Metab. Res..

[34] Weigand I., Knobloch L., Flitsch J., Saeger W., Monoranu C.M., Höfner K., Herterich S., Rotermund R., Ronchi C.L., Buchfelder M. (2019). Impact of USP8 Gene Mutations on Protein Deregulation in Cushing Disease. J. Clin. Endocrinol. Metab..

[35] Roosterman D. (1997). Endocytosis of the Rat Somatostatin Receptors: Subtype Discrimination, Ligand Specificity, and Delineation of Carboxy-Terminal Positive and Negative Sequence Motifs. DNA Cell Biol..

[36] Hukovic N., Panetta R., Kumar U., Patel Y.C. (1996). Agonist-Dependent Regulation of Cloned Human Somatostatin Receptor Types 1-5 (HSSTR1-5): Subtype Selective Internalization or Upregulation. Endocrinology.

[37] Albani A., Perez-Rivas L.G., Tang S., Simon J., Lucia K.E., Colón-Bolea P., Schopohl J., Roeber S., Buchfelder M., Rotermund R. (2022). Improved Pasireotide Response in USP8 Mutant Corticotroph Tumours in Vitro. Endocr. Relat. Cancer.

[38] Carr H.S., Zuo Y., Frost J.A. (2023). The Wnt Pathway Protein Dvl1 Targets Somatostatin Receptor 2 for Lysosome-Dependent Degradation. J. Biol. Chem..

[39] Ren J., Jian F., Jiang H., Sun Y., Pan S., Gu C., Chen X., Wang W., Ning G., Bian L. (2018). Decreased Expression of SFRP2 Promotes Development of the Pituitary Corticotroph Adenoma by Upregulating Wnt Signaling. Int. J. Oncol..

[40] Liu X., Feng M., Dai C., Bao X., Deng K., Yao Y., Wang R. (2019). Expression of EGFR in Pituitary Corticotroph Adenomas and Its Relationship with Tumor Behavior. Front. Endocrinol..

[41] Hao H.X., Jiang X., Cong F. (2016). Control of Wnt Receptor Turnover by R-Spondin-ZNRF3/RNF43 Signaling Module and Its Dysregulation in Cancer. Cancers.

[42] Mukai A., Yamamoto-Hino M., Awano W., Watanabe W., Komada M., Goto S. (2010). Balanced Ubiquitylation and Deubiquitylation of Frizzled Regulate Cellular Responsiveness to Wg/Wnt. EMBO J..

[43] Madana B., Walkerb M.P., Young R., Quick L., Orgel K.A., Ryan M., Gupta P., Henrichc I.C., Ferrer M., Marine S. (2016). USP6 Oncogene Promotes Wnt Signaling by Deubiquitylating Frizzleds. Proc. Natl. Acad. Sci. USA.

[44] Tauriello D.V.F., Maurice M.M. (2010). The Various Roles of Ubiquitin in Wnt Pathway Regulation. Cell Cycle.

[45] Liu J., Xiao Q., Xiao J., Niu C., Li Y., Zhang X., Zhou Z., Shu G., Yin G. (2022). Wnt/β-Catenin Signalling: Function, Biological Mechanisms, and Therapeutic Opportunities. Signal Transduct. Target. Ther..

[46] Srivastava A., Rikhari D., Srivastava S. (2024). RSPO2 as Wnt Signaling Enabler: Important Roles in Cancer Development and Therapeutic Opportunities. Genes. Dis..

[47] Yue F., Ku A.T., Stevens P.D., Michalski M.N., Jiang W., Tu J., Shi Z., Dou Y., Wang Y., Feng X.-H. (2024). Loss of ZNRF3/RNF43 Unleashes EGFR in Cancer. bioRxiv.

[48] Nieman L.K., Biller B.M.K., Findling J.W., Newell-Price J., Savage M.O., Stewart P.M., Montori V.M., Edwards H. (2008). The Diagnosis of Cushing’s Syndrome: An Endocrine Society Clinical Practice Guideline. J. Clin. Endocrinol. Metab..

[49] Fleseriu M., Auchus R., Bancos I., Ben-Shlomo A., Bertherat J., Biermasz N.R., Boguszewski C.L., Bronstein M.D., Buchfelder M., Carmichael J.D. (2021). Consensus on Diagnosis and Management of Cushing’s Disease: A Guideline Update. Lancet Diabetes Endocrinol..

[50] Bertagna X., Guignat L., Groussin L., Bertherat J. (2009). Cushing’s Disease. Best. Pract. Res. Clin. Endocrinol. Metab..

[51] Liu C., Lo J.C., Dowd C.F., Wilson C.B., Kunwar S., Aron D.C., Tyrrell J.B. (2004). Cavernous and Inferior Petrosal Sinus Sampling in the Evaluation of ACTH-Dependent Cushing’s Syndrome. Clin. Endocrinol..

[52] Teramoto A., Nemoto S., Takakura K., Sasaki Y., Machida T. (1993). Selective Venous Sampling Directly from Cavernous Sinus in Cushing’s Syndrome. J. Clin. Endocrinol. Metab..

[53] Graham K.E., Samuels M.H., Nesbit G.M., Cook D.M., O’Neill O.R., Barnwell S.L., Loriaux D.L. (1999). Cavernous Sinus Sampling Is Highly Accurate in Distinguishing Cushing’s Disease from the Ectopic Adrenocorticotropin Syndrome and in Predicting Intrapituitary Tumor Location. J. Clin. Endocrinol. Metab..

[54] Chen E.Y., Tan C.M., Kou Y., Duan Q., Wang Z., Meirelles G.V., Clark N.R., Ma’ayan A. (2013). Enrichr: Interactive and Collaborative HTML5 Gene List Enrichment Analysis Tool. BMC Bioinform..

