# Supplementary figures and images for: Cushing’s Disease Manifestation in USP8-Mutated Corticotropinoma May Be Mediated by Interactions Between WNT Signaling and SST Trafficking
Source: Int J Mol Sci. 2024 Nov 29;25(23):12886. doi: 10.3390/ijms252312886 (PMC11641039; doi:10.3390/ijms252312886)

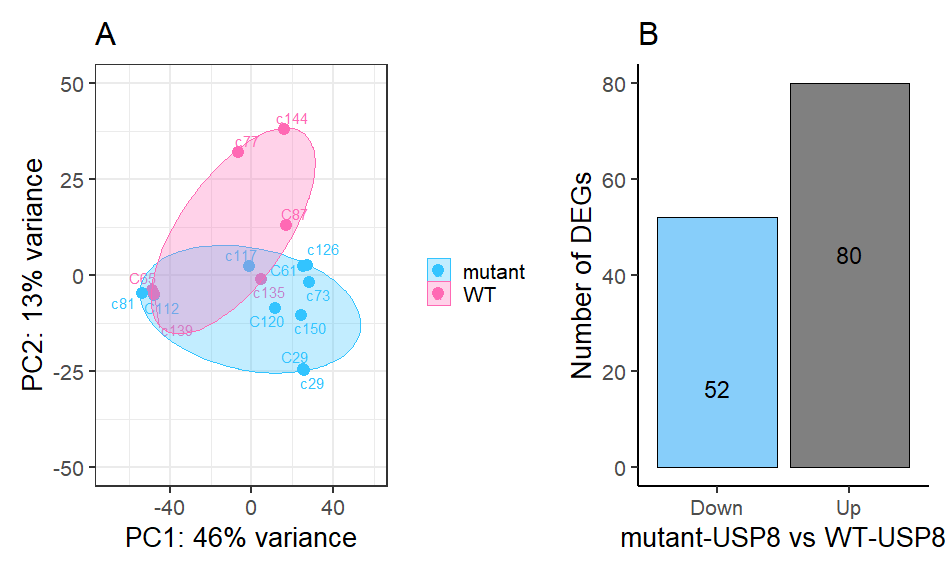

Supplement: Supplementary file 1 [file ijms-25-12886-s001.zip › Figure S1.png]
